# Soyasaponin II protects against acute liver failure through diminishing YB-1 phosphorylation and Nlrp3-inflammasome priming in mice

**DOI:** 10.7150/thno.40128

**Published:** 2020-02-03

**Authors:** Fangzhao Wang, Shenhai Gong, Teng Wang, Lei Li, Haihua Luo, Junhao Wang, Chenyang Huang, Hongwei Zhou, Guiming Chen, Zhanguo Liu, Qifan Zhang, Yong Jiang, Peng Chen

**Affiliations:** 1 Department of Pathophysiology, Guangdong Provincial Key Laboratory of Proteomics, School of Basic Medical Sciences, Southern Medical University, Guangzhou, China,; 2 State Key Laboratory of Organ Failure Research; Southern Medical University, Guangzhou, China,; 3 Department of Intensive Care Unit, Zhujiang Hospital, Southern Medical University, Guangzhou, China.; 4 Department of Hepatobiliary Surgery, Nanfang Hospital, Southern Medical University, Guangzhou, China,; 5 Microbiome Medicine Center, Zhujiang Hospital, Southern Medical University, Guangzhou, China.

**Keywords:** acute liver failure, soyasaponin II, Nlrp3-inflammasome, YB-1

## Abstract

Acute liver failure is characterized by the rapid development of liver dysfunction and remarkably high mortality. Accumulating evidence suggests that soyasaponin possesses potential anti-inflammatory activities. Here, we aimed to investigate the potential role of soyasaponin II in acute liver failure and establish the underlying mechanism.

**Methods**: Lipopolysaccharide/D-galactosamine (LPS/GalN) was employed to induce acute liver failure. We applied liquid chromatography and mass spectrometry (LC/MS) to characterize the changes of soyasaponin II levels in the cecal content and liver. Transcriptomics and proteomics analysis were used to evaluate the functional molecule mediated by soyasaponin II in macrophages.

**Results**: LPS/GalN administration markedly decreased fecal and hepatic soyasaponin II levels. Soyasaponin II treatment protected mice against LPS/GalN induced acute liver injury. Additionally, soyasaponin II markedly diminished Y-Box Binding Protein 1 (YB-1) phosphorylation and nuclear translocation, Nlrp3 inflammasome priming, and interleukin 1β (Il-1β) production in macrophages. Phosphorylated YB-1 could activate Nlrp3 mRNA transcription by binding the promoter region. Finally, immunofluorescence analysis showed elevated p-YB-1 nuclear translocation in macrophages of acute liver failure patients compared to controls.

**Conclusion**: Our data shows that soyasaponin II which serves as a novel inhibitor for YB-1 phosphorylation and Nlrp3 inflammasome priming could protect mice against LPS/GalN induced acute liver failure.

## Introduction

Acute liver failure (ALF) is a severe and complicated clinical condition, and patients can deteriorate sharply when the innate immune system is amplified inappropriately 1. High morbidity and mortality is associated with multiple etiologies including virus hepatitis, acetaminophen overdose, indeterminate elements, and Wilson disease (hepatolenticular degeneration) 2-3. Of note, the pathogenesis of ALF includes not only direct liver damage but immunologically mediated processes that are commonly triggered by various causes 4-5. However, effective strategies for identifying pharmacologic targets in order to ameliorate the excess inflammation in ALF are still needed.

Recent prominent research efforts have advanced our understanding of the role of inflammasome in ALF 6-8. For example, Ilyas et al reported that Il-1β overproduction due to inflammasome activation in macrophages is recognized as the main contributor for ALF in preclinical model 9. Nonetheless, the underlying molecule associated with the activation of inflammasome in ALF remains elusive. Because inflammasome as an intermediary agent contributes to such severe immunoreaction as previously reported 10-12, it is imperative to understand its elaborate regulatory mechanism.

Mammalian Y-box binding protein 1 (YB-1) is a versatile protein which contains an evolutionarily conserved cold-shock domain (CSD), binds DNA/RNA sequences preferentially, and orchestrates transcription as either a repressor or activator 13. Previous studies pointed to YB-1 as possibly relevant to the apoptotic pathway, DNA damage recognition, and gene repair, leading to variety of unfavorable outcomes such as sarcoma invasion, metastasis, and cancer drug resistance 14-16. It has also been reported that AKT phosphorylates YB-1 at Ser102 located in the cold shock domain, which affects the growth of breast cancer cells 17. Notably, YB-1 also showed increased expression in certain inflammatory diseases 18-19, but its role in the innate immune system is still equivocal.

Legumes in our diet contain many compounds, but soyasaponin A and B are the most prolific phytochemicals in soybeans 20. It is increasingly feasible to take advantage of soyasaponins for their salutary and bioactive anti-virus, antioxidant, and anti-carcinogenic characteristics, but it is their anti-inflammatory properties that attracted our attention 20-21. This study aims to thoroughly explore the influence of a certain type of soyasaponin (soyasaponin II, SSII) on the immunological alterations during endotoxin induced acute liver failure, as well as to search for a pivotal molecule that is mechanically dependent on this nutrient in order to identify a potential clinical therapeutic target.

## Materials and Methods

### Animal model

Eight- to ten-week-old male C57BL/6J mice were used. Mice were given oral administration of soyasaponin II (SSII; ChromaDex, 5 mg/kg dissolved in dimethyl sulfoxide (DMSO)) once a day for three consecutive days. Control animals received the equivalent volume of DMSO. On the third day, the mice received intraperitoneally administered D-galactosamine (GalN; Macklin, 700 mg/kg) in combination with Lipopolysaccharide (LPS, isotype O111:B4; Sigma-Aldrich, 10 μg/kg). 6 h after the LPS/GalN injection, the mice were sacrificed for the following experiments. For acetaminophen overdose experiment, mice were pretreated with or without SSII for three days and received intraperitoneal injection with acetaminophen (300 mg/kg, dissolved in PBS). After 24 h, the mice were sacrificed for the following experiments. All experimental procedures were in accordance with the National Institutes of Health guidelines and were approved by the local Animal Care and Use Committee of the Southern Medical University.

### Soyasaponin II analysis

Cecal content and liver were collected and a liquid chromatography-mass spectrometry (LC-MS) system was used to analyze SSII levels. Water was added to the sample at 9-fold volume/weight and extracted ultrasonically for 10 min, then methanol was added and the samples were centrifuged at 13000 rpm for 10 min at 4 °C. The supernatant was concentrated by drying under nitrogen, and then dissolved into mobile phase for analysis by HPLC. Chromatographic separation was conducted on a Thermo Scientific Prelude SPLC system, and Thermo TSQ Vantage triple quadrupole mass spectrometer was used for detection. The chromatographic column was Hypersil gold 50*2.1 mm, 1.9 μm. Mobile phase A was comprised of a 0.1% formic acid water solution and mobile phase B was methanol. The gradient elution was carried out according to the following procedure: 0-1.25 min, 90% A and 10% B; 1.25-2.25 min, 20% A and 80% B; 2.25-5.25 min, 5% A and 95% B; 5.25-6.25 min, 90% A and 10% B. Data acquisition and analysis were performed with TraceFinder^TM^ software version 3.3 sp1 (Thermo Fisher Scientific Corp., USA).

### Transcriptome analysis

The RNA from hepatic macrophages was extracted and RNA quality was detected by NanoDrop 2000. High-quality RNA were used for cDNA libraries construction and sequencing (Wuhan Bioacme Technologies Corp.). RNA sequencing libraries were generated using the Kapa RNA library prep kit for illumina with multiplexing primers, according to the manufacturer's protocol. Then, we performed sequencing on an Illumina NovaSeq sequencer to obtain raw data. The reference genome (GRCm38.94) was used for quality controlled raw data, and quantitation of gene expression were conducted. We employed R package (ClusterProfiler/enrichplot) to perform the enrichment analysis of the KEGG signaling pathway by GSEA (Gene Set Enrichment Analysis) method as described previously 22-23. Briefly, we first calculate log_2_ fold change (DMSO/SSII) between the two groups of all genes expression. Then, GSEA was carried out to analyze the differences between two groups (DMSO/SSII). The gene sets were referring to KEGG (Kyoto Encyclopedia of Genes and Genomes) database. The ridgeplot will visualize expression distributions of core enriched genes for GSEA enriched categories. The parameters used in the pre-ranked GSEA were the classic enrichment statistic, with 1,000 gene set permutations, and a false discovery rate (FDR) <0.25 was recognized as statistically significant.

### Proteomics Analyses

Proteins of isolated hepatic macrophages in SSII treated or non-treated group (n=10) were mixed. The sample was prepared as describe previously 24. In brief, according to the standard filter-aided sample preparation (FASP) protocol, the protein sample was mixed with 8 mol/L urea and centrifuged. After reduction by dithiothreitol (DTT), alkylation was performed with iodoacetamide (IAA). Then trypsin was added to digest the proteins into peptides. Data independent acquisition (DIA)-based quantitative proteomic analysis 25 was performed by Orbitrap Fusion™ Tribrid™ connected with EASY-nLC1200 (Thermo Scientific, Waltham, MA, USA). Triplicate analyses were performed for both groups (DMSO and SSII). Identified proteins with a P value<0.05 and a fold change (FC) greater than 1.5 compared to the control group were considered significantly dysregulated. GO annotation was implemented to explore the gene regulatory networks (http://www.geneontology.org). Kyoto Encyclopedia of Genes and Genomes (KEGG, http://www.genome.jp/kegg/) pathway enrichment analysis was performed to investigate the significant pathways enriched by the differentially expressed genes.

For further details regarding the materials used, please refer to the supplementary information.

## Results

### ALF reduced intestinal and hepatic soyasaponin II levels in mice

**Figure 1:**
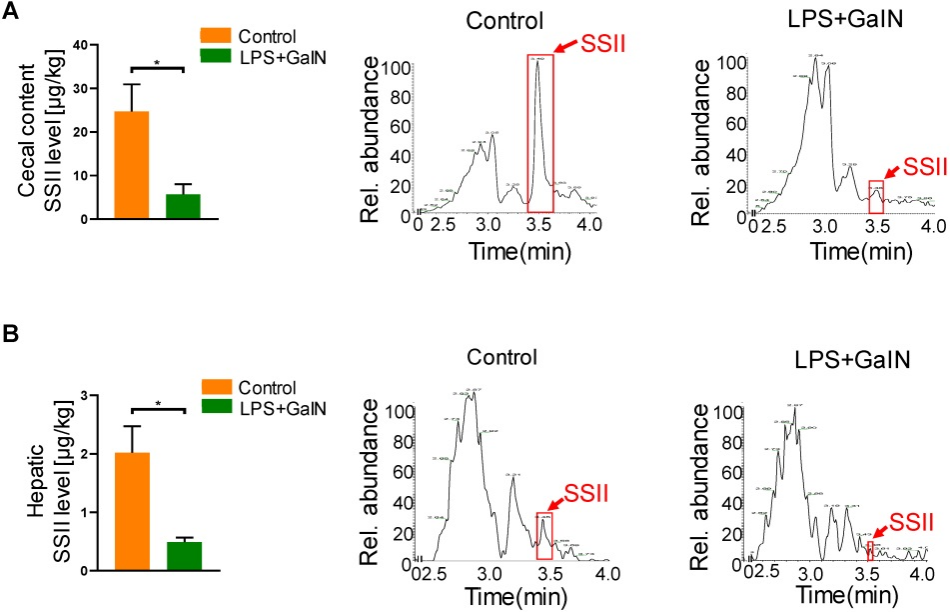
** Acute liver failure occurrence reduced intestinal and hepatic soyasaponin II levels in mice.** C57BL/6J mice were intraperitoneally administrated with D-galactosamine (700 mg/kg) in combination with lipopolysaccharide (10 μg/kg). Cecal contents and liver were collected after LPS/GalN treatment for 6 h. (A) Soyasaponin II level in cecal content and the representative chromatogram (n=5). (B) Soyasaponin II levels in liver and the representative chromatogram (n=3 for LPS/GalN-treated and n=4 for PBS-treated mice). *p<0.05.

Soyasaponin II (SSII) is a bioactive material extracted from leguminous plants, which has a wide range of health-promoting effects. In order to explore the biological role of SSII during LPS/GaIN induced acute liver failure pathogenesis, we first performed LC/MS analysis to compare the level of SSII between LPS/GaIN administrated mice and control mice. As shown in Figure 1, SSII levels in the cecal content and liver were significantly lower in the LPS/GaIN group than the control group. As known, the presence of soyasaponins *in vivo* is dependent on diet supplementation with soybean foods. In order to rule out the possibility that different food intake contributed to the different levels of soyasaponin II in mice between control with LPS/GaIN group, we monitored food consumption after LPS/GaIN treatment. As shown in Figure S1, we found that the difference of food consumption was not statistically significant. Thus, these results also manifested that the difference of SSII between LPS/GalN treated and control group is not due to the food intake and we speculated that SSII may play an important role in LPS/GaIN induced acute liver failure.

### Soyasaponin II pretreatment mitigated ALF

**Figure 2:**
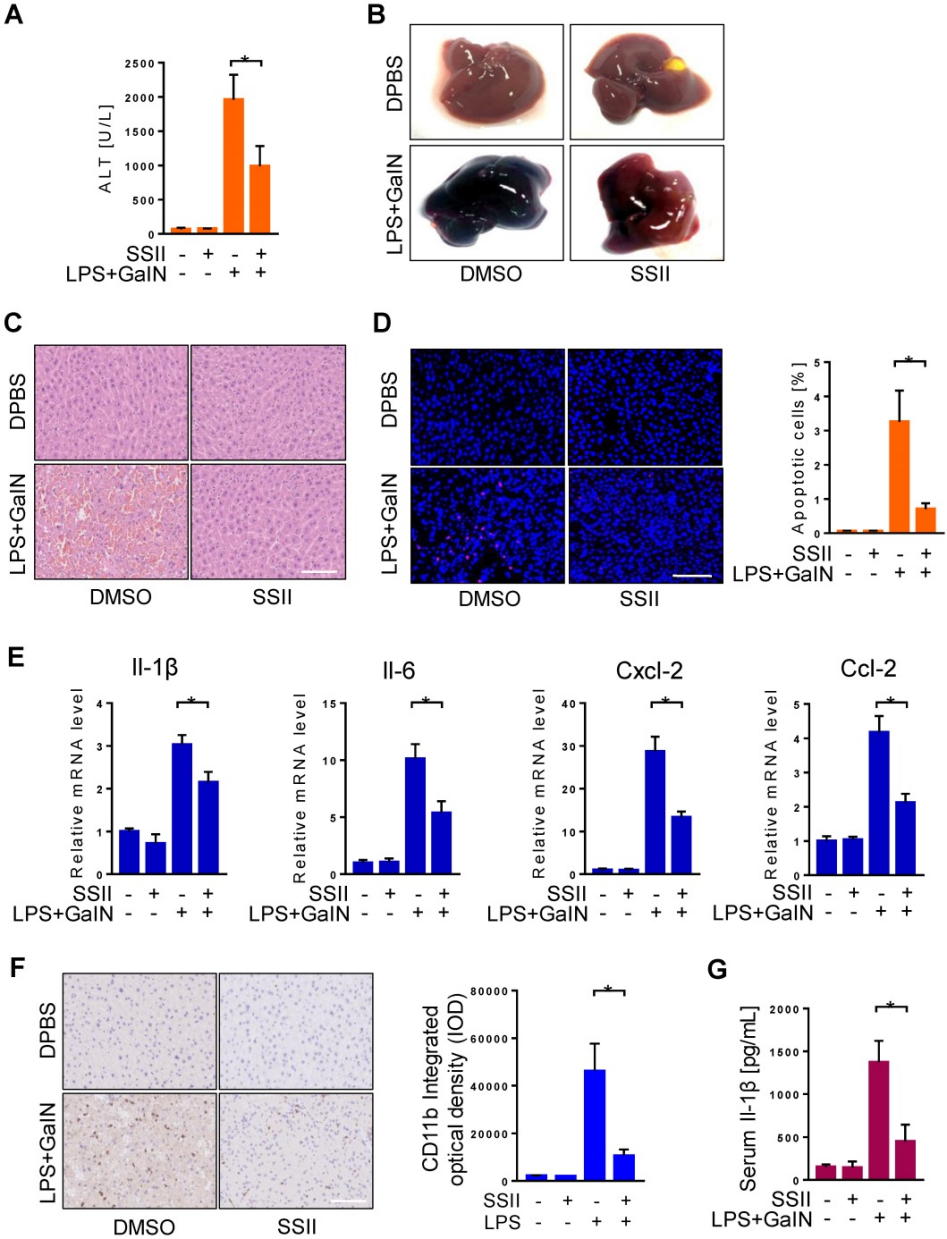
** Soyasaponin II pretreatment mitigated acute liver failure.** After pretreatment with or without soyasaponin II (5 mg/kg) for 3 days, mice were intraperitoneally administered D-galactosamine (700 mg/kg) in combination with lipopolysaccharide (10 μg/kg) for 6 h. (A) Effect of SS II (5 mg/kg, i.g) pretreatment on ALT levels (n=14-15 for LPS/GalN-treated and n=4 for PBS-treated mice). (B) Representative photographs of livers. (C) HE staining and (D) TUNEL staining of liver samples. The number of apoptotic cells was quantified (n=5 for LPS/GalN-treated and n=4 for PBS-treated mice). (E) qPCR analyses of hepatic Il-1β, Il-6, Cxcl-2, Ccl-2 gene expression. (n=8 for LPS/GalN treated and n=4 for PBS treated mice). (F) Immunohistochemical staining for CD11b in liver sections (n=5-7 for LPS/GalN-treated and n=3 for PBS-treated mice). (G) Level of serum Il-1β (n=8-9 for LPS/GalN treated and n=4 for PBS treated mice). Scale bars: 100 μm. *p<0.05.

To further investigate the function of SSII in LPS/GalN induced ALF, mice received pretreatment with or without SSII to compare their response. We firstly found soyasaponin II levels were higher in the liver and cecal content after soyasaponin II administration (Figure S2A-2B), suggesting soyasaponin II could be uptake by liver. Furthermore, after LPS/GalN, compared to the control mice, plasma alanine aminotransferase (ALT) levels in SSII pretreated mice were remarkably decreased (Figure 2A). Liver morphology, HE staining, and TUNEL staining confirmed the ALT results (Figure 2B-D). Local inflammatory response in the liver was indicated by increased mRNA levels of inflammatory cytokines, including Il-1β, Il-6, Cxcl-2, Cxcl-10, Ccl-2, Ccl-4, Ccl-7 (Figure 2E, Figure S2C), the mRNA levels of these inflammatory factors were reduced in SSII-pretreated mice compared to control mice. Then, we detected CD11b expression level by immunohistochemistry to assess monocyte and neutrophil recruitment in the liver. As expected, we found that LPS/GaIN treatment significantly increased the number of CD11b+ cells in the liver, whereas SSII pretreatment reduced CD11b+ cells recruitment (Figure 2F). Systematic inflammatory response was demonstrated by elevated levels of serum Il-1β (Figure 2G). Together, these results, consistent with previous studies 26, demonstrate that SSII pretreament exhibited protective effects against LPS/GalN-induced ALF.

We post-treated SSII 1 hour after LPS/GalN to check whether post-treatment could alleviate acute liver injury *in vivo*. Unfortunately, plasma alanine aminotransferase (ALT) levels in SSII post-treated mice exhibited decrease trend without significance compared with control mice, furthermore hepatic HE staining confirmed the ALT results (Figure S3A-B). These data indicated post-treatment may be less efficient than pre-treatment. Additionally, SSII administration could not attenuate acetaminophen (APAP) induced liver damage as evidenced by similar plasma ALT level and hepatic histologic changes between two groups (Figure S3C-D), suggesting SSII pretreament did not protect APAP induced acute liver failure.

### Soyasaponin II ameliorated canonical Nlrp3 inflammasome-associated innate immune response

**Figure 3:**
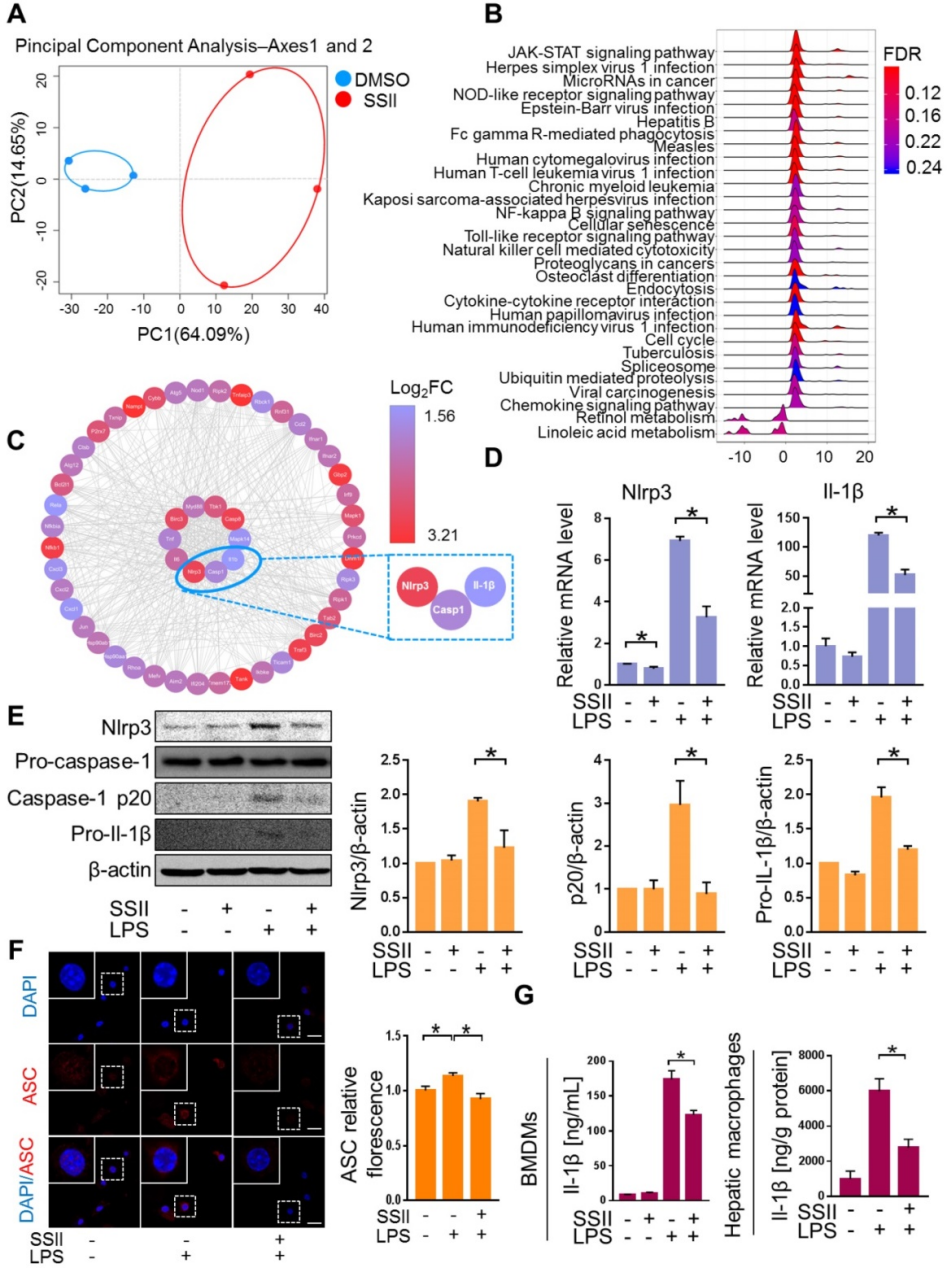
** Soyasaponin II ameliorated canonical Nlrp3 inflammasome-associated innate immune response.** After pretreatment with or without soyasaponin II (5 mg/kg) for 3 days, mice were intraperitoneally administered D-galactosamine (700 mg/kg) in combination with lipopolysaccharide (10 μg/kg) for 6 h. Hepatic macrophages were isolated, followed by transcriptome analysis. (A) Scatter plots of PCA of hepatic macrophage gene expression profile (n=3). (B) KEGG signaling pathway by GSEA (n=3). (C) Hub gene analysis of NOD-like receptor signaling pathway by CytoHubba (n=3). (D) Mature BMDMs were stimulated with DPBS or LPS for 2 h, with or without SSII (5 μg/ml) co-incubation. The relative mRNA levels of Nlrp3 and Il-1β (n=4). (E) Representative western blot analysis for Nlrp3, caspase-1 (precursor and spliced variant) and pro-Il-1β from BMDMs cell lysis solution and the quantification results (n=4). (F) Representative immunofluorescence images of ASC in BMDMs, and the fluorescence quantification results. Red represents ASC; Blue represents DAPI (n=3). (G) Cleaved Il-1β released in BMDMs culture supernatant was quantified by ELISA (n=10 for LPS-treated and n=8 for PBS-treated cells) and Il-1β levels from isolated liver macrophages (n=4-5 for LPS/GalN-treated and n=3 for PBS-treated mice). Scale bars: 20 μm. *p<0.05.

We next explored the underlying mechanism for the protective effects of SSII. Macrophages were found to play a pivotal role in ALF development 27-29. To screen for the key molecule mediated by SSII, we performed transcriptome analysis of the macrophages isolated from the liver after LPS/GaIN exposure treatment with or without soyasaponin II. PCA analysis showed that the gene-expression profile of soyasaponin II-stimulated hepatic macrophages had significant differences when compared to controls after LPS/GaIN treatment (Figure 3A, Figure S4A). GSEA (Gene Set Enrichment Analysis) highlighted NOD-like receptor signaling as a top ten enriched Kyoto Encyclopedia of Genes and Genomes (KEGG) pathway that decreased visibly after LPS/GaIN exposure treatment in the soyasaponin II group (Figure 3B, Figure S4B). We next selected NOD-like receptor signaling pathway-related genes for hub gene analysis and found that inflammasome related genes, such as Nlrp3 and Il-1β, were mainly influenced upon SSII administration (Figure 3C).

Accumulating evidence described the pivotal role of the Nlrp3 inflammasome in ALF. Consequently, based on the above transcriptome results, we speculated that SSII may have an antagonistic effect on Nlrp3 inflammasome activity. Bone marrow-derived macrophages (BMDMs) were challenged with LPS in the absence or presence of SSII. As expected, Nlrp3 and Il-1β displayed decreased gene expression levels in the SSII group compared to the control group (Figure 3D). Next, we compared the responsiveness of Nlrp3 inflammasome protein levels to LPS-stimulated BMDMs in both groups. The Nlrp3, caspase-1 (p20), and pro-Il-1β expression after LPS stimulated significantly decreased when incubated with SSII (Figure 3E). SSII group indicated less ASC accumulation compared with SSII non-treated group, indicating SSII also attenuated Nlrp3 inflammasome activation (Figure 3F). Given the fact that Il-1β serves as a key contributor to ALF, we measured cleaved Il-1β in BMDMs supernatant by ELISA. We confirmed that SSII substantially blocked the release of mature Il-1β (Figure 3G). Decreased hepatic macrophage Il-1β protein levels in mice after SSII treatment were also found (Figure 3G). Taken together, these findings corroborate the theory that SSII decreases the immune reaction by abolishing Nlrp3 inflammasome component priming, and that this inhibitory activity is believed to be actuated by a transcriptional program.

### Soyasaponin II blocked YB-1 phosphorylation in macrophages

**Figure 4:**
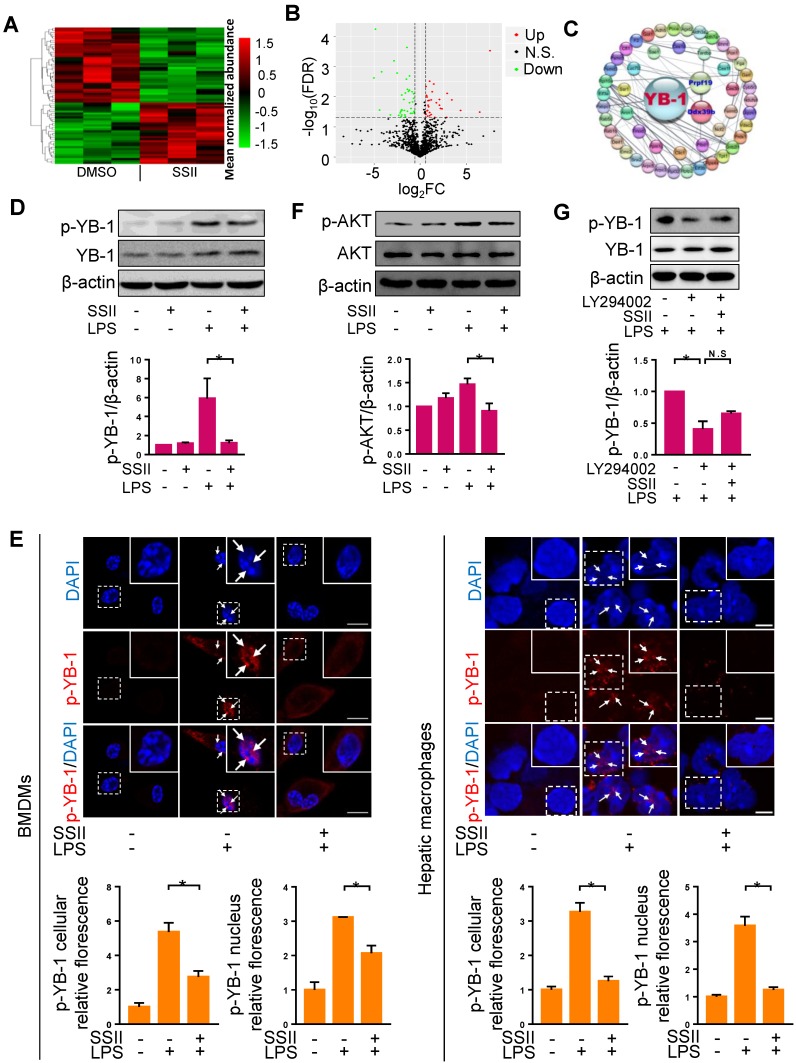
** Soyasaponin II blocked YB-1 activation in macrophages.** (A, B) Heat-Map and volcano plots of liver macrophages proteins with protein cluster analysis of differentially regulated proteins in DMSO and SSII groups from LPS/GaIN treated animals. Plot of SSII/DMSO ratios (Log_2_FC) versus -Log_10_FDR of liver macrophage proteins: green dots represent downregulated proteins, red dots represent differentially up-regulated proteins. (C) Protein-protein interaction (PPI) network diagram of discrepant proteins connected with YB-1 from the two groups. (D) Mature BMDMs were stimulated with PBS or LPS for 2 h, with or without co-incubation of SSII (5 μg/ml), and cells harvested after activation with ATP (5mM) for 30min. Representative western blot analysis for phosphorylated YB-1 and total YB-1 in cell lysis solution is displayed, as well as the protein quantification (n=4). (E) Left panel: PBS or LPS primed BMDMs with (n=3) or without (n=3) co-incubation with SSII (5 μg/ml) in petri dishes for 2 h. Immunofluorescence staining with p-YB-1 antibody and the fluorescence quantification. Right panel: Macrophages isolated from LPS/GaIN treated mouse liver with (n=3) or without (n=3) SSII pretreatment for three days. Immunofluorescence staining with p-YB-1 antibody and the fluorescence quantification. (F) BMDMs were stimulated with PBS or LPS for 15 min, with or without co-incubation of SSII (5 μg/ml). Representative western blot for phosphorylated AKT in BMDMs and the quantification results (n=3). (G) BMDMs was pretreated with LY294002 (10 μM) for 30 min, then were stimulated with LPS co-treatment with or without soyasaponin II. Representative western blot for phosphorylated YB-1 and the quantification results (n=3). Scale bars: 10 μm. *p<0.05.

Based on the potential interaction between soyasaponin II and Nlrp3 inflammasome, we sought to further decipher this molecular pathway by employing mass spectrometry-based proteomics analysis. A panoramic view by unsupervised hierarchical clustering analysis of the relative quantitative data showed that the composition and abundance of proteins in liver macrophages in ALF mice pre-conditioned with soyasaponin II, had greatly changed compared with controls (Figure 4A). Volcano Plot analysis showed that noteworthy proteins subdivided into two major categories based on the enrichment of differential proteins (Figure 4B). Next, we conducted gene ontology (GO) enrichment analyses to interpret the idiographic function of the discrepant protein sequences biologically. According to the -Log_10_ (FDR) and the observed gene count, proteins belonging to Biological Process, Cellular Component and Molecular Function showed differences between SSII treated and non-treated groups (Figure S5A). As expected, according to (KEGG) pathway, there was obvious alteration in protein catabolism (such as valine, leucine, and isoleucine degradation, etc.) processes, reflecting the differences of the liver macrophages between the two groups (Figure S5B). Two proteins exhibited significantly different trend: PRPF19 (Reactome database identifier: R-MMU-5420907) which presented the highest fold change, and Ddx39B (Reactome database identifier: R-MMU-8849139) which also showed significant alteration were noted. We ultimately took a crack at deciphering the discrepancy by looking for the senior protein which regulates these two proteins. Surprisingly, by searching the Reactome database and using the protein-protein interaction (PPI) network, we found these two proteins were potentially interconnected with YB-1 (Figure 4C), suggesting that YB-1 may be responsible for driving the different pathophysiologic phenotype between control and SSII groups.

YB-1 is a transcription factor which can be activated by phosphorylation, and YB-1 nuclear translocation plays an important role in diseases development 30. Consistent with previous studies 31, we found that YB-1 primarily expresses in the cytoplasm under normal status (Figure S6A). We then reasoned that YB-1 phosphorylation regulates YB-1 nuclear translocation. To verify this hypothesis, we transfected pcDNA-YB-1 mutant (Ser^102^ to Glu^102^, p-YB-1 overexpression) plasmid or pcDNA-YB-1 plasmid (control) to macrophages, followed by LPS treatment. We first confirmed that p-YB-1 was overexpressed in pcDNA-YB-1 mutant (Ser^102^ to Glu^102^) plasmid transfected cells (Figure S6B), and as shown in Figure S6C, we found that YB-1 nuclear translocation was significantly increased in pcDNA-YB-1 glutamic acid mutant plasmid group compared to control group. These results suggested that YB-1 phosphorylation can directly promote YB-1 nuclear translocation. We next attempted to monitor whether phosphorylated YB-1 levels were influenced by SSII. Western blotting revealed that the level of phosphorylated YB-1 was markedly elevated in BMDMs after LPS administration, whereas SSII significantly mitigated p-YB-1 compared to controls (Figure 4D). To further detail the inhibitory effects of SSII on phosphorylated YB-1 levels in nucleus, we performed immunofluorescence staining for both BMDMs and mice liver macrophages. As presented in Figure 4E, LPS challenge promoted p-YB-1 nuclear translocation, but treatment with SSII largely prevented this critical protein from phosphorylating and transportation into the nucleus. Emerging evidence has indicated that AKT phosphorylates YB-1 at Ser102 in the cold shock domain 17. Here, studies were conducted to determine whether soyasaponin II regulates YB-1 phosphorylation (Ser102) through mediating AKT activation or not. As shown in Figure 4F, AKT was activated upon LPS challenge compared to control group and soyasaponin II treatment markedly downregulated AKT phosphorylation in BMDMs. Furthermore, BMDMs were pretreated with LY294002 which is an inhibitor of AKT signaling, and then were stimulated with LPS co-treated with or without soyasaponin II. As shown in Figure 4G, we found that LY294002 markedly decreased YB-1 phosphorylation level whereas soyasaponin II treatment did not further reduce YB1 phosphorylation in BMDMs. Therefore, we concluded that soyasaponin II reduced YB-1 phosphorylation after LPS challenge may be mediated by AKT signaling.

### The repression of Nlrp3 inflammasome by soyasaponin II is dependent on YB-1

**Figure 5:**
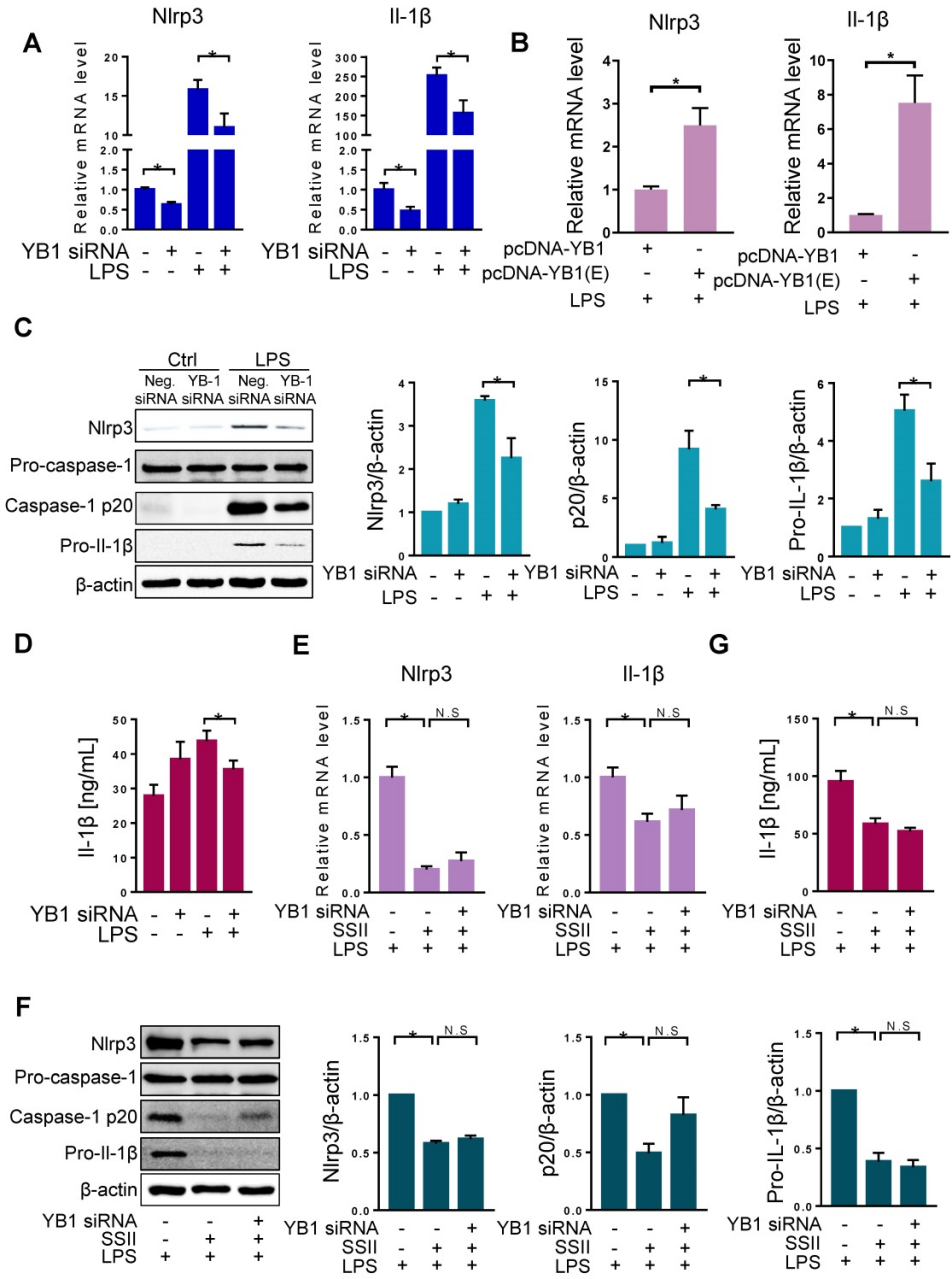
** The repression of Nlrp3 inflammasome by soyasaponin II was dependent on YB-1.** (A) BMDMs were transfected with YB-1 si-RNA plasmid or negative control (NC) plasmid for 36 h, then incubation with LPS or PBS for 2 h. The relative mRNA levels of Nlrp3 (n=12 for LPS-treated and n=4 for PBS-treated cells) and Il-1β (n=12-13 for LPS-treated and n=8-9 for PBS-treated cells). (B) BMDMs were transfected with pcDNA-YB-1 mutant (Ser102 to Glu102, p-YB-1 overexpression) plasmid or pcDNA-YB-1 (control) plasmid for 36 h, followed by LPS treatment for 2 h. The relative mRNA levels of Nlrp3 and Il-1β mRNAs (n=8). (C) BMDMs were transfected with YB-1 si-RNA plasmid or negative control (NC) plasmid for 36 h, then incubation with LPS or PBS for 2 h. Western blot of Nlrp3, pro-caspase-1, caspase-1 p20 and pro-Il-1β, as well as the quantification results (n=3). (D) Cleaved Il-1β released in cell supernatant were quantified by ELISA (PBS group n=4, LPS group n=20). (E) LPS-primed BMDMs were co-incubated with or without SSII for 2 h, transfected with or without YB-1 si-RNA for 36 h, and the relative expression mRNA levels of Nlrp3 (n=5 for YB-1 si-RNA-treated and n=6-10 for negative control (NC) plasmid -treated cells) and Il-1β (n=7). (F) Western blot of Nlrp3, pro-caspase-1, caspase-1 p20 and pro-Il-1β, as well as the quantification results (n=5). (G) Cleaved Il-1β released in cell supernatant were quantified by ELISA (n=8). *p<0.05.

Since SSII can impact both YB-1 and Nlrp3 inflammasome priming, we next investigated the molecular correlation between YB-1 and Nlrp3 inflammasome. First, we designed YB-1 siRNA and confirmed the repression effect (Figure S7). YB-1 mRNA knockdown markedly lowered the mRNA levels of Nlrp3 and Il-1β in BMDMs after exposure to LPS (Figure 5A). Moreover, as shown in Figure 5B, we found higher mRNA levels of Nlrp3 and Il1β in the BMDMs transfected with pcDNA3.1-YB-1 glutamic acid mutant plasmid (phosphor-mimetic mutant of YB-1) compared with pcDNA3.1-YB-1 plasmid. Furthermore, destabilization of YB-1 mRNA showed decreased protein levels of Nlrp3, cleaved caspase-1, and pro-Il-1β (Figure 5C). Also, secreted Il-1β in BMDMs was markedly diminished in the YB-1 siRNA group after LPS challenge (Figure 5D). These experiments indicate that YB-1 signaling attenuates Nlrp3 inflammasome priming and the release of pro-inflammatory cytokine Il-1β.

Our data shows that both SSII and YB-1 knockdown could repress Nlrp3 inflammasome. To further link these molecules, we set up three independent groups: LPS, LPS + SSII, and LPS + SSII + YB-1 siRNA. Figure 5E-G shows that in the SSII supplemented microenvironment, Nlrp3 inflammasome priming was sharply decreased compared with the LPS group, but when co-treated with YB-1 siRNA, the degree of canonical Nlrp3 inflammasome activation was equivalent to the group without siRNA. These data demonstrate that SSII suppression of the LPS-stimulated Nlrp3 inflammasome priming may depend on YB-1 in macrophages.

### YB-1 binds the promoter region of Nlrp3 to upregulate transcriptional activity

**Figure 6:**
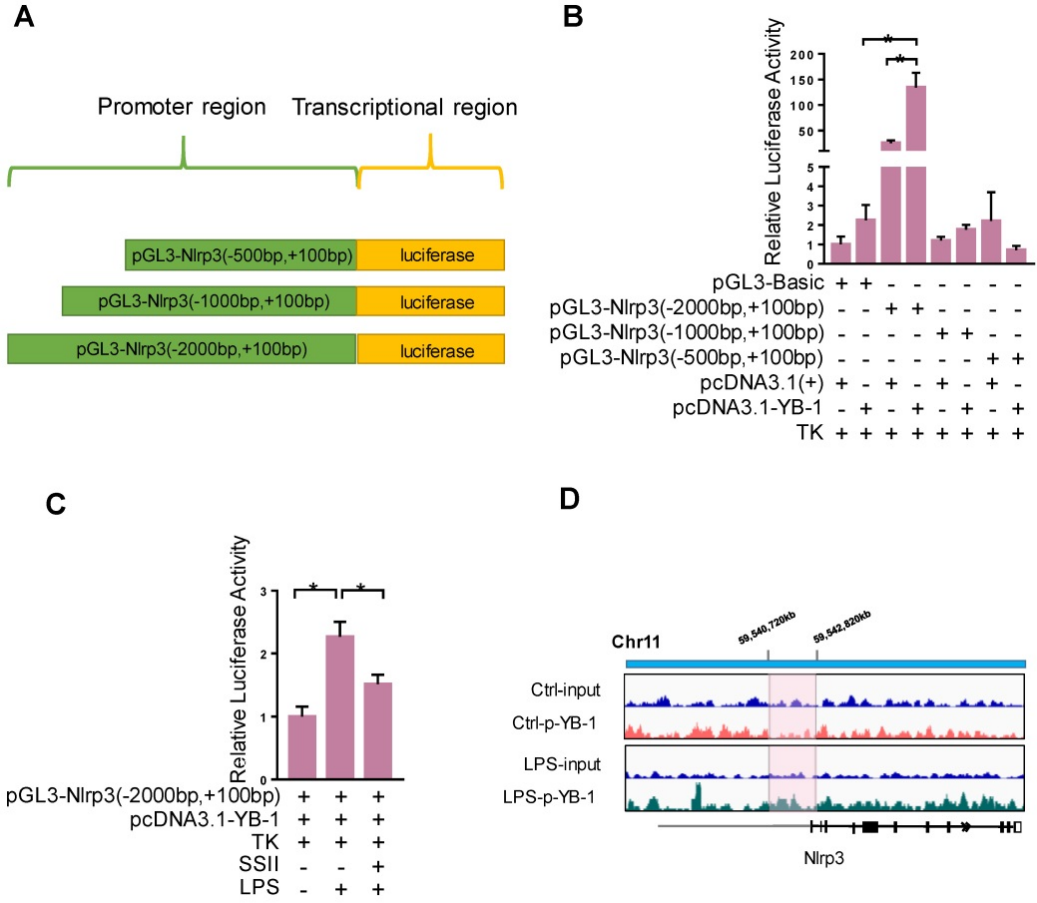
** YB-1 binds the promoter region of Nlrp3 to upregulate transcriptional activity.** (A) The sketch map of plasmid. (B) Raw264.7 cells were transfected with pGL3-promoter reporter plasmid and pcDNA3.1-YB-1 plasmid or pcDNA3-basic plasmid for 36 h, followed by LPS treatment for 2 h. Relative luciferase activity in each group (n=4). (C) Raw264.7 cells were transfected with pGL3-promoter reporter plasmid and pcDNA3.1-YB1 plasmid for 36 hours. Then Raw264.7 cells were treated with or without SSII for 2 h in presence of LPS. Relative luciferase activity in each group (n=5-7 for LPS-treated and n=5 for PBS-treated cells). (D) The chromatin from Raw264.7 cells that were treated with or without LPS (500 ng/ml) for 2 h was collcted for the immunoprecipitation by using p-YB-1 antibody. DNA was extracted and sequenced. Integrative Genomics Viewer (IGV) at locus containing Nlrp3 was shown. *p<0.05.

We further explored the detailed molecular mechanism by which YB-1 stimulates Nlrp3 at the transcription level. We speculate that YB-1 protein directly binds the promoter region of Nlrp3 and regulates its expression. To confirm this, the Dual-Luciferase Report Gene System was used. Figure 6A-6B showed that pcDNA3.1-YB-1 plasmid dramatically increased the luciferase activity of pGL3-Nlrp3 (-2000bp to +100bp promoter region) compared to pcDNA3-basic plasmid in macrophage. In addition, the luciferase activity of pGL3-Nlrp3 promoter reporter plasmid was increased upon LPS challenge compared to control group, and soyasaponin II treatment markedly suppressed the luciferase activity (Figure 6C). Furthermore, as shown in Figure 6D, ChIP sequencing results confirmed that p-YB-1 could bind to the promoter region of Nlrp3 gene in macrophage under LPS stimulation. These results suggest that YB-1, specifically p-YB-1 can interact with Nlrp3 promoter to upregulate transcription.

### Phosphorylated YB-1 is enriched in the macrophage nuclei of ALF patients

**Figure 7:**
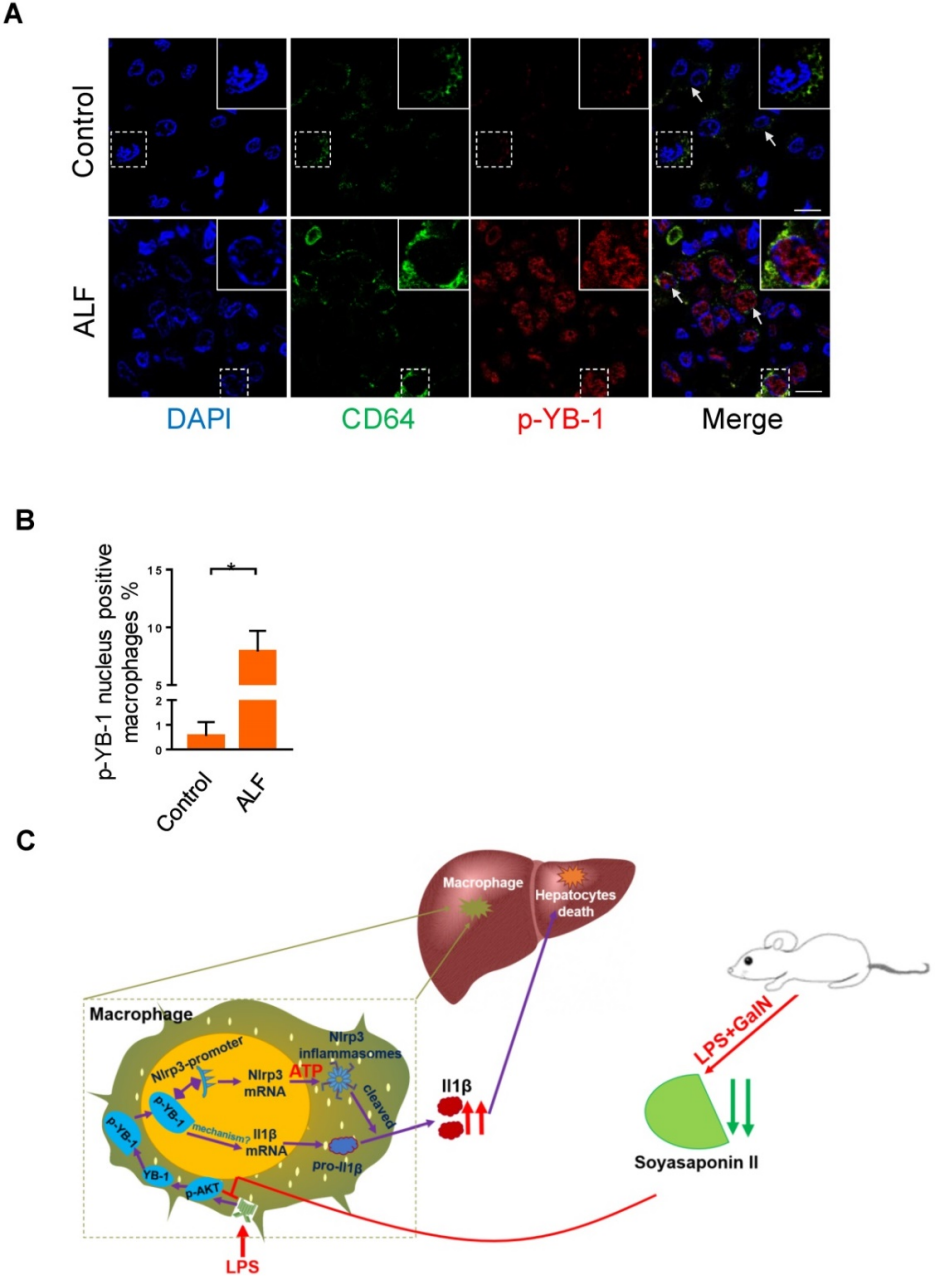
** Phosphorylated YB-1 was enriched in the macrophage nuclei of ALF patients.** (A, B) Macrophages in human liver paraffin section marked by CD64 antibody, and p-YB-1 expression were detected by immunofluorescence, and the p-YB-1 nuclear translocation positive macrophages were quantified (control n=5; ALF n=11). (C) Working model: LPS/GalN challenge decreased fecal and hepatic soyasaponin II levels. Soyasaponin II repressed YB-1 phosphorylation and nuclear translocation, p-YB-1 could translocate into nuclear and bind the promoter of Nlrp3, to promote Nlrp3 inflammasome priming, Il-1β overproduction and liver damage. Scale bars: 10 μm. *p<0.05.

Our above data clearly showed that p-YB-1 may be a key regulator in murine liver failure, we finally linked evidence from mouse models to data from ALF patients. Total YB-1 was mainly located in cytosol in control liver under normal circumstance (Figure S8), while immunofluorescence staining of liver sections from ALF patients showed statistically significant increases in p-YB-1 in the nuclei of CD64 marked macrophages compared with controls (Figure 7A-B). This confirmed the presence of increased p-YB-1 in the liver macrophages of ALF patients.

## Discussion

ALF is characterized as a rapidly deteriorating disease with different etiologies causing overwhelming hepatocyte death including systemic inflammatory response syndrome 1-3. Clinical information and tissue samples collected and dissected by US Acute Liver Failure Study Group indicate that focusing on inflammasome may lead to some therapeutic discoveries for treatment of ALF 6. In this regard, new treatment methods should be established pressingly. Epidemiological investigations show that soybean products contain essential amino acids, unsaturated fatty acids, dietary fiber, minerals and a variety of phytochemicals, which are considered to be benefit for human and animals 32. Although there are no direct clinic evidences to show eating more soybeans could protect from ALF, it is reported that black soybean could modulate cholesterol metabolism and mitigate oxidative damage during non-alcoholic fatty liver disease progression 33. Besides, the preclinical studies also demonstrated that administration of soyasaponins, extracted from soybeans, effectively protected mice from alcohol induced acute hepatotoxicity 34. These conclusions provide strong support for soyasaponins research in acute or chronic liver diseases, but it remains need to be confirmed in clinical trial. Our research investigated the anti-inflammation role of SSII and its protective mechanisms in LPS/GaIN induced ALF. With regards to clinical therapy, another objective of this study was to discover an intermediary mediator so as to interpret the integrated signal pathway in acutely inflamed liver disease. It has been reported that Nlrp3 inflammasome complex plays an important role in ALF 35-37. Our data further shows that soyasaponin II protects mice against LPS/GaIN-induced acute liver injury by depressing Nlrp3 inflammasome priming to reduce Il-1β release. This finding provides a new treatment method for acute liver injury in the preclinical model, but more clinical research is needed to investigate the role of soyasaponin II in treating ALF. Whether or not soyasaponins can be absorbed by the intestinal tract has been a focus of debate. Early studies found few soyasaponins uptake by the circulatory system, and soyasaponins are not detected in the blood or urine of hosts who have been exposed to a soybean diet 38-40. It was generally believed that soyasaponins were hydrolyzed into metabolites by the gut microbiota and then excreted in the feces. However, other recent studies found that soyasaponins could be detected in human and rat serum using high-performance liquid chromatography-tandem mass spectrometry (HPLC-MS/MS) and that the bioavailability of group B soyasaponins was better than group A 41-42. We also successfully detected soyasaponin II in the liver with LC/MS. In conclusion, it is reasonable to speculate that a certain amount of soyasaponin II can be absorbed into liver after soyasaponin II treatment and exert an anti-inflammatory effect by suppressing Nlrp3 inflammasome priming in ALF progression.

It is reported that certain types of intestinal microbiota including Lactobacillus, Bifidobacteria, Escherichia and Bacteroides can degrade saponins by synthetizing β-glucosidase and/or other glycosides metabolizing enzymes 43. Hence, we hypothesized that changes in the composition and function of the gut microbiota may lead to the altered levels of soyasaponin II in LPS/GaIN induced ALF mice. In order to verify the hypothesis, we performed metagenomics of cecal contents to analyze gut microbiota in the control and LPS/GalN groups. There was a change in the intestinal microbial composition of control group compared with the LPS/GaIN group (Figure S9A). Then, we conducted a functional analysis of the identified gut microbiota using CAZy (Carbohydrate-Active enZYmes Database) mapping. We found a difference in glycosyl transferases which mediates the metabolic processing of glycosides compound 44, suggesting the difference in the function of the gut microbiota to metabolize glycosides between control and LPS/GaIN group (Figure S9B). Our data preliminarily concluded that the reduction of intestinal and hepatic soyasaponin II levels in LPS/GalN treated mice may be partially resulted from altered gut microbial composition and function. Future research is needed to clarify the specific mechanisms.

Empirical studies have linked YB-1 to the epithelial-to-mesenchymal transition (EMT) that is associated with breast carcinoma aggressiveness, suggesting YB-1 signaling could mediate the metastasis of epithelial malignancies. Apart from its oncogenic role, our work shows that YB-1 induces inflammation by binding to the transcriptional initial region of Nlrp3. Although we found YB-1 could also mediate Il-1β mRNA level in macrophages, there was no difference of Il-1β promoter luciferase activity between pcDNA3.1-YB-1 plasmid compared to pcDNA3.1-basic plasmid (data not shown), indicating the altered gene expression of Il-1β observed in YB-1 treated group may be mediated by indirect manner, for example, YB-1 could interact with other important transcription factor in the nuclear and promoter Il-1β transcription. Future work is needed to specify the detailed mechanism. However, our current results strongly suggest that YB-1 could function as potential therapy for treating inflammsome-mediated inflammatory diseases. It has been reported that heterozygous YB-1 knockout reduced hepatic chemokine/chemokine expression and diminished granulocyte infiltration into the liver, and these YB-1 knockout mice were protected from LPS induced mortality compared to WT mice 45. The role of inflammsome has been studied in many types of liver diseases, and it is thought to be a primary contributor to hepatocyte injury, immunological cell motivation, and hepatic inflammation. Further investigation is needed to determine whether YB-1 has a similar effect on other inflammasomes such as NLRP1, NLRC4, IPAF, AIM2, etc. Additionally, our data revealed that soyasaponin II is a novel inhibitor for YB-1 activation, implying that it may be beneficial for other pathologic processes mediated by YB-1.

Macrophages orchestrate multiple missions in immune system, but the excessive activation of immune cells may give rise to the tissue injury. Based on our findings related to the inflammation-suppressive effects of SSII, coupled with the ability of this soy compound to manipulate key transcription factor, we theorize that the inflammatory response of YB-1 in macrophages is mitigated by SSII in ALF (Figure 7C). Our results confirm the molecular immunological role of SSII, and we believe SSII effect on YB-1 could be clinically applicable for treating hepatic diseases.

## Supplementary Material

Supplementary materials and methods, figures, and tables.
