# Supplementary material for: Soyasaponin II protects against acute liver failure through diminishing YB-1 phosphorylation and Nlrp3-inflammasome priming in mice
Source: Theranostics. 2020 Feb 3;10(6):2714–26. doi: 10.7150/thno.40128 (PMC7052911; doi:10.7150/thno.40128)
Supplement: Supplementary file 1 — Supplementary materials and methods, figures, and tables. [file thnov10p2714s1.pdf]

Supplementary materials and methods

Patient samples

Informed consent acquired by all patients who fulfilled criteria for ALF and control individuals. The controls were adjacent normal liver tissues harvested from patients underwent surgery for liver cancer. The experimental design and study were approved by the Institutional Ethical Committee. Detail information about ALF patients were shown in Table S1.

Aminotransferases analysis

Plasma alanine aminotransferase (ALT) was assessed using commercial kits (Nanjing Jiancheng Bioengineering Institute) according to manufacturer’s instructions.

Histological analysis

Mice livers were fixed in 10% formalin (Biosharp), then stained with hematoxylin and eosin. For immunohistochemistry experiment, CD11b antibody (Abcam) was used according to standard protocol. The terminal deoxynucleotidyl transferase-mediated deoxyuridine triphosphate nick end-labeling (TUNEL) assay was used to assess cells apoptosis with a commercial kit (KeyGene) according to the manufacturer’s protocol.

Hepatic macrophage isolation

The hepatic macrophage isolation was described previously [1-2]. In brief, mice livers were collected, minced, and digested with collagenase type IV (Sigma-Aldrich), Pronase E (Solarbio) and DNase I (Sigma-Aldrich) at 37 ℃ for 20 min, followed by centrifuging at 800g for 5 min at 4 ℃, and then discard

the supernatant carefully. Resuspend cell pellet with ice-cold PBS (BSA 0.5%). Then centrifuge at 800g for 5 min at 4 ℃. Resuspend the cells pellet in 6 ml of 40% Percoll (sigma-Aldrich) and pipet the cell suspension on 2 ml of 80% Percoll (sigma-Aldrich). The nonparenchymal cell population were isolated by centrifuging at 800g for 30 min at 4 ℃ without brake. The harvest cell ring was collected and cultured in RPMI-1640 supplemented with 20% FBS and 1% Penicillin-Streptomycin solution. After 2 h, the adherent cells were collected for experiments.

Murine bone marrow-derived macrophage immortalization

Bone marrow-derived macrophages (BMDMs) were harvested after growth from bone marrow progenitor cells obtained from femurs and tibias of male C57BL/6J mice. MSCF (Macrophage Colony Stimulating Factor, Recombinant Mouse M-CSF Protein, 416-ML-050, RnDsystems, 20 ng/ml) was used to select macrophages. Cells were cultured with DMEM medium coupled with 10% fetal bovine serum and 1% Penicillin-Streptomycin at 37 °C incubator. For LPS/ATP challenge, differentiated BMDMs were cultured for 24 h first. Then macrophages were primed with 500 ng/ml LPS for 2 h to activate Nlrp3 inflammasome as well as its downstream pathway, and incubated with 50 mM ATP (Adenosine 5′-triphosphate disodium salt solution, A6559-25UMO, Sigma) for another 30 min with the purpose of secreting IL-1β. For SSII treatment, the macrophages were co-incubated with SSII (5 μg/ml) and LPS (500 ng/ml) for 2 h, whereas the control group was treated with a same concentration of DMSO.

ASC detection

After primed with LPS for 2 h and ATP for 30 min, BMDMs, seeded on petri dish, fixed with 4% paraformaldehyde (PFA) for 15 min at room temperature and washed with DPBS three times. A

permeabilization procedure and a block process are followed. Then cells were incubated with anti-ASC antibody (sc-514414, Santa Cruz) overnight at 4 °C in a final dilution of 1:200. For quantification of ASC, six to ten random fields were selected and the relative mean immunofluorescence intensity was measured using Image J.

Transfection of small interfering RNA

Cells were fasted by serum-free medium. Then BMDMs were transfected with nonspecific control siRNA or mouse YB-1 siRNA nucleotide fragment (GenePharma) using the LipofectaminTM RNAiMAX (Invitrogen) for 36 h. Target primer sequences for RNAi knockdown of mouse YB-1 (Gene ID: 22608): (5'-3') CCACGCAAUUACCAGCAAATT (sense) and (5'-3') UUUGCUGGUAAUUGCGUGGTT (antisense). Negative control (NC) primer sequences: (5'-3') UUCUCCGAACGUGUCACGUTT (sense) and (5'-3') ACGUGACACGUUCGGAGAATT (antisense). YB-1 phosphor-mimetic mutant plasmid (Ser^{102} to Glu^{102}) was designed commercially (Genemei Biotech, Guangzhou).

Dual-Luciferase Reporter Gene System

For Nlrp3 promoter study, Raw264.7 were cultured in 24-well plates (2.5×10^5/well) and transfected with 0.375 μg each of pGL3-promoter reporter plasmid (Genemei Biotech, Guangzhou) or equivalent amounts of empty pGL3-basic plasmid and 37.5 ng TK plasmid. Meanwhile, Raw264.7 cells were co-transfected with 0.375 μg pcDNA3.1-YB-1 expression plasmid or empty pcDNA3.1 vector. After cell transfection for 36 h, followed by LPS challenge for 2 h, Raw264.7 cells were lysed and the supernatant was evaluated for luciferase activity using the Dual-Luciferase Reporter Assay System (Promega) according to the manufacturer’s instructions.

ChIP-seq analysis

Raw264.7 cells were cultured in 15 cm dish and treated with or without LPS for 2 h. Then cells were fixed with formaldehyde. The chromatin from Raw264.7 cells was digested into 150-900 bp DNA/protein fragments by using SimpleChIP® Plus Enzymatic Chromatin IP Kit (9500, CST) according to the manufacturer's instructions. And the immunoprecipitation was employed by using p-YB-1 antibody (2900, CST). After purification, the precipitated DNA and input DNA was analyzed by sequencing.

Immunofluorescence staining

BMDMs placed on a petri dish were stimulated with LPS. Liver macrophages were placed on a microslide. After the dewaxing procedure, the sections were soaking with 1×modified sodium citrate antigen repair solution (Beyotime, P0083). Then the samples were incubated in microwave on high (100%) for 5 min, heat (40%) for 5 min, followed by a ceasefire for 3 min, and the samples were cooled to room temperature. Then after the fixation in 4% Paraformaldehyde (PFA) for 15 min at room temperature, the samples were gently lavaged with PBS every 5 min three times. After permeabilization with 0.1% Triton X-100 solution for 5-10 min, the samples were blocked with DPBS containing 3% BSA for 1 h. Excess buffer was drained, and the tissue sections were washed three times by DPBS. The coverslip was placed to allow a certain amount of p-YB-1 antibody (bs-3477R-AF594, Bioss) to disperse over the entire section and the samples were incubated in a moist chamber overnight at 4°C. For counterstaining, DAPI staining was performed for 5 min at room temperature, and then washed slides three times. The images were captured with Zen software for LSM 880 laser Airscanning microscope. Relative mean fluorescence was analyzed with Fiji image J software, randomly greater than or equal to 5 views of each petri dish or section were selected to

conduct the quantitive analysis. For human samples, greater than or equal to 5 fields of per slice were selected to calculate the average of the ratio of CD64/p-YB-1 double-stained positive cells to the total number of cells.

Immunoblot analysis

BMDM cell lysates were soaked in ice-cold RIPA buffer in the presence of PMSF and the Halt™ Phosphatase Inhibitor Single-Use Cocktail (Halt™ Protease Inhibitor Cocktail, Thermo). Equivalent proteins in each lane were electrophoretically separated by SDS-polyacrylamide gel electrophoresis (PAGE), transferred to nitrocellulose membranes, and blotted with the following primary antibodies: Nlrp3 (15101S, CST), Caspase-1 (AG-20B-0042-C100, AdipoGen), IL-1β (31202S, CST), p-YB-1 (A8481, Assay Biotech), YB-1 (4202S, CST), and β-actin (3700S, CST), p-AKT (4060, CST), AKT (2920, CST).

Metagenomics analysis

Cecal content DNA of PBS or LPS/GalN treated group (n=3) were mixed and metagenomics analysis was performed as previously described [3-4]. In brief, the microbial DNA was randomly fragmented followed by end repair and A tailing, adapter ligation, post-ligation cleanup, library amplification and post-amplification cleanup. The DNA sample was sequenced using the Illumina/MiSeq sequencing platform and then raw data acquisition by base calling. In order to improve the quality of information analysis, the raw data filtering was conducted with Trimmomatic v0.36. The CAZy (Carbohydrate-Active enZYmes Database) analysis was used for gene ontology.

Cytokine ELISA

IL-1β protein levels were determined by commercial kit (Neobioscience) based on manufacturer's instructions.

Quantitative real-time PCR analysis

Following RNA extraction from cells or liver using Trizol agent according to the manufacturer's instructions, a reverse transcription (RT) procedure was performed to synthesize complementary DNA (cDNA) using a reverse transcription kit (TOYOBO). Then real-time polymerase chain reaction (PCR) quantification was performed using SYBR® Green Realtime PCR Master Mix (TOYOBO). All primers are listed in Supplementary Table 2. The $2^{(-\triangle\triangle C(T))}$ was used to analyze the relative quantification and 18S was used to normalize expression data.

Statistical analysis

The data were presented as mean ± standard error of the mean (SEM). The two-tailed Student’s t-test was used. The significance level was $p < 0.05$.

Table S1. Clinical information of acute liver failure

Parameter	ALF	Ctrl
**Number of patients**	11	5
**Aetiology**	*Drug-induced*	n/a
	Mixed overdose	
	Non-drug induced	
	Buddi-Chiari	
	Hepatitis B	
**Bilirubin(umol/L)**	453.87	n/a
	[250.00-938.54]	
**ALT(IU/mL)**	137.79	n/a
	[57.10-462.00]	
**AST(IU/mL)**	171.08	n/a
	[77.00-498.00]	
**CTP**	11.22	n/a
	[8.00-14.00]	
**MELD**	33.30	n/a
	[24.00-40.00]	

Abbreviations:

ALF: acute liver failure
Ctrl: control
AST: aspartate aminotransferase;
ALT: alanine aminotransferase;
CTP: Child-Turcotte-Pugh;
MELD: Model for End Stage Liver Disease

Table S2. Primers for qPCR

	Left primer(5'-3')	Right primer(5'-3')
**18s**	CGATCCGAGGGCCTCACTA	AGTCCCTGCCCTTTGTACACA
**Il-6**	TGATGCACTTGCAGAAAACA	ACCAGAGGAAATTTTCAATAGGC
**Il-1β**	GGTCAAAGGTTTGGAAGCAG	TGTGAAATGCCACCTTTTGA
**Ccl-2**	CCTGCTGTTCACAGTTGCC	ATTGGGATCATCTTGCTGGT
**Ccl-4**	CATGAAGCTCTGCGTGTCTG	GAAACAGCAGGAAGTGGGAG
**Ccl-7**	CTGCTTTCAGCATCCAAGTG	TTCCTCTTGGGGATCTTTTG
**Cxcl-2**	CGGTCAAAAAGTTTGCCTTG	TCCAGGTCAGTTAGCCTTGC
**Cxcl-10**	CTCATCCTGCTGGGTCTGAG	CCTATGGCCCTCATTCTCAC
**Nlrp3**	ATTACCCGCCCGAGAAAGG	TCGCAGCAAAGATCCACACAG

Supplementary reference

1. Pourcet B, Zecchin M, Ferri L, Beauchamp J, Sitaula S, Billon C, et al. Nuclear Receptor Subfamily 1 Group D Member 1 Regulates Circadian Activity of NLRP3 Inflammasome to Reduce the Severity of Fulminant Hepatitis in Mice. Gastroenterology. 2018; 154: 1449-1464.e20.

2. Sebti Y, Ferri L, Zecchin M, Beauchamp J, Mogilenko D, Staels B, et al. The LPS/D-Galactosamine-Induced Fulminant Hepatitis Model to Assess the Role of Ligand-Activated Nuclear Receptors on the NLRP3 Inflammasome Pathway In Vivo. Methods Mol Biol. 2019; 1951: 189-207.

3. Abubucker S, Segata N, Goll J, Schubert AM, Izard J, Cantarel BL, et al. Metabolic Reconstruction for Metagenomic Data and Its Application to the Human Microbiome. PLoS Comput Biol. 2012; 8: e1002358.

4. Franzosa EA, Morgan XC, Segata N, Waldron L, Reyes J, Earl AM, et al. Relating the metatranscriptome and metagenome of the human gut. Proc Natl Acad Sci U S A. 2014 3; 111: E2329-38.

Supplementary figures

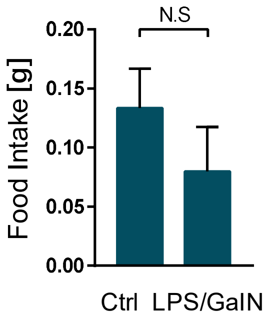

Figure S1. LPS/GalN did not significantly affect food intake in mice. C57BL/6J mice were intraperitoneally administrated with D-galactosamine (700 mg/kg) in combination with lipopolysaccharide (10 μg/kg). The food intake with or without LPS/GalN treatment for 6 h (n=3 for PBS treated and n=5 for LPS/GalN treated mice).

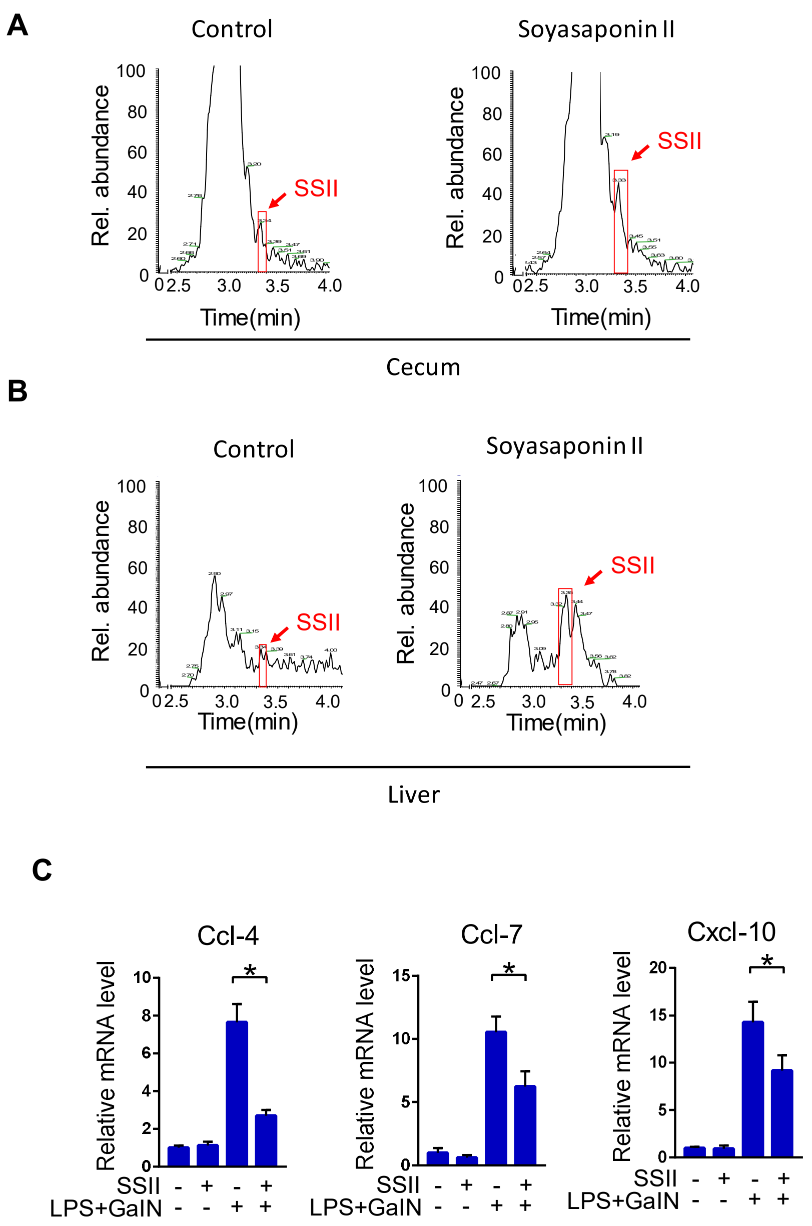

Figure S2. Soyasaponin II was increased after SSII treatment and SSII alleviated hepatic chemokines overexpression after LPS/GalN. (A-B) Mice were orally administrated with SSII (5 mg/kg) for 3 days. The representative chromatogram of SSII in cecal content and liver. (C) After pretreatment with or without soyasaponin II (5 mg/kg) for 3 days, mice were intraperitoneally administered D-galactosamine (700 mg/kg) in combination with lipopolysaccharide (10 μg/kg) for 6 h. qPCR analyses of hepatic Ccl-4, Ccl-7, Cxcl-10 gene expression. (n=8 for LPS/GalN treated and n=4 for PBS treated mice). *$p<0.05$.

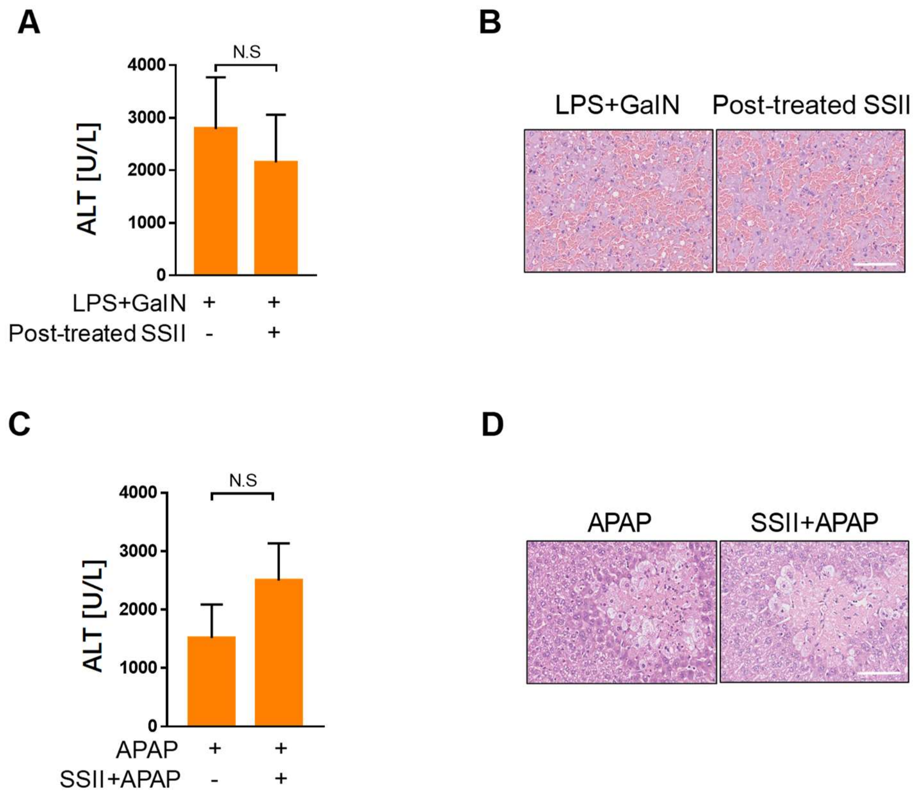

Figure S3. Post-treatment of SSII did not mitigate ALF and the effect of SSII on acetaminophen induced liver injury. C57BL/6J mice were post-treated SSII (5 mg/kg) 1 h after LPS/GalN administration. (A) Plasma ALT level (n=4 for LPS/GalN treated and n=5 for SSII post treatment mice. (B) HE staining of livers. (C) After pretreatment with or without soyasaponin II (5 mg/kg) for 3 days, mice were intraperitoneally administered APAP (300 mg/kg) for 24 h. Effect of SS II (5 mg/kg) treatment on ALT levels (n=6-8). (D) HE staining of livers. Scale bars: 100 μm.

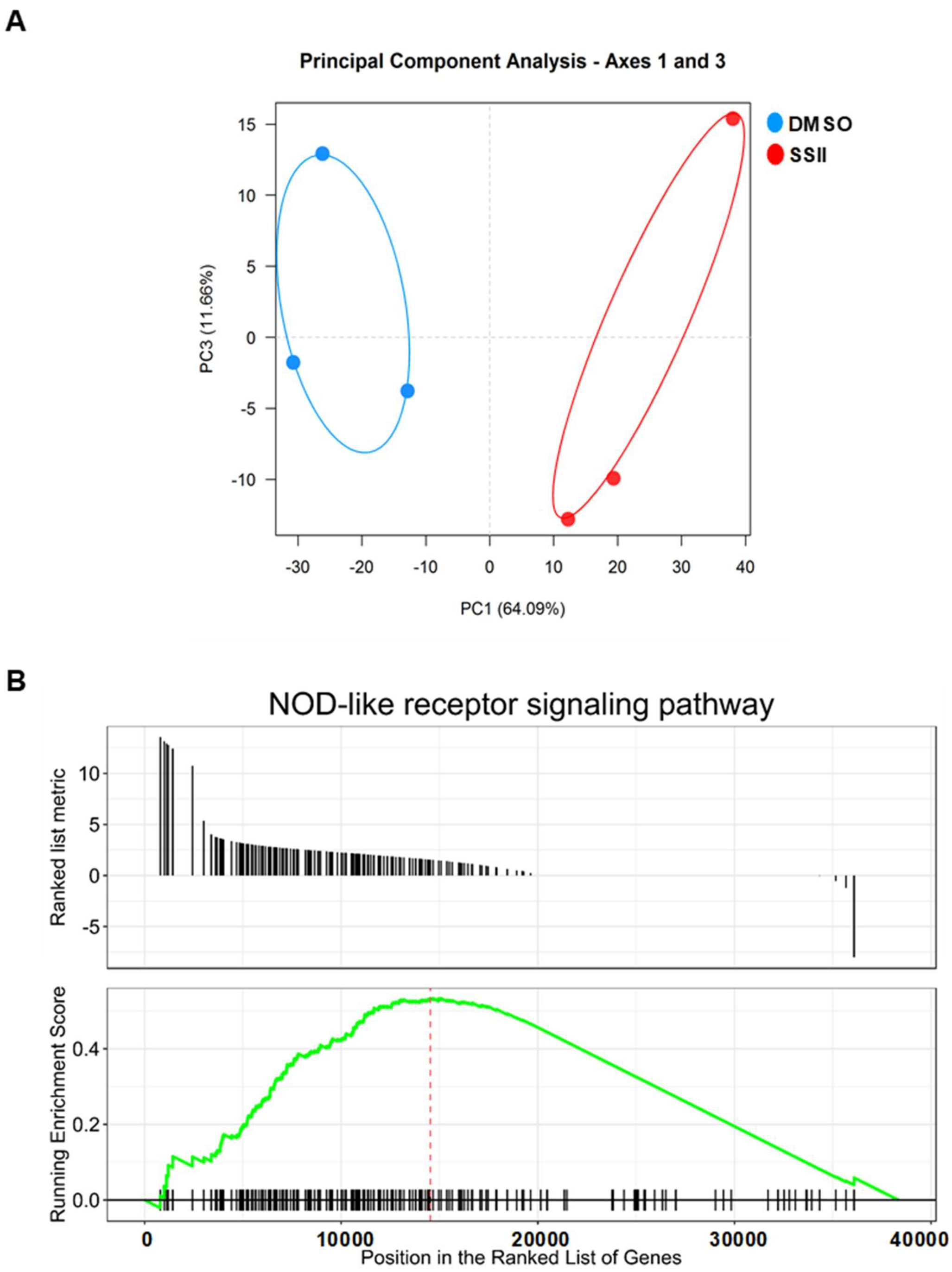

Figure S4. Soyasaponin II altered gene expression profile in macrophages after LPS/GalN. After pretreatment with or without soyasaponin II (5 mg/kg) for 3 days, mice were intraperitoneally administered D-galactosamine (700 mg/kg) in combination with lipopolysaccharide (10 μg/kg) for 6 h. Then mice hepatic macrophage was isolated for transcriptome analysis. (A) Scatter plots of PCA of hepatic macrophage gene expression in control and LPS/GalN group. (B) The NOD-like receptor signaling

pathway from GSEA enrichment analysis. Running Enrichment Score depends on related gene fold change (DMSO/SSII).

A

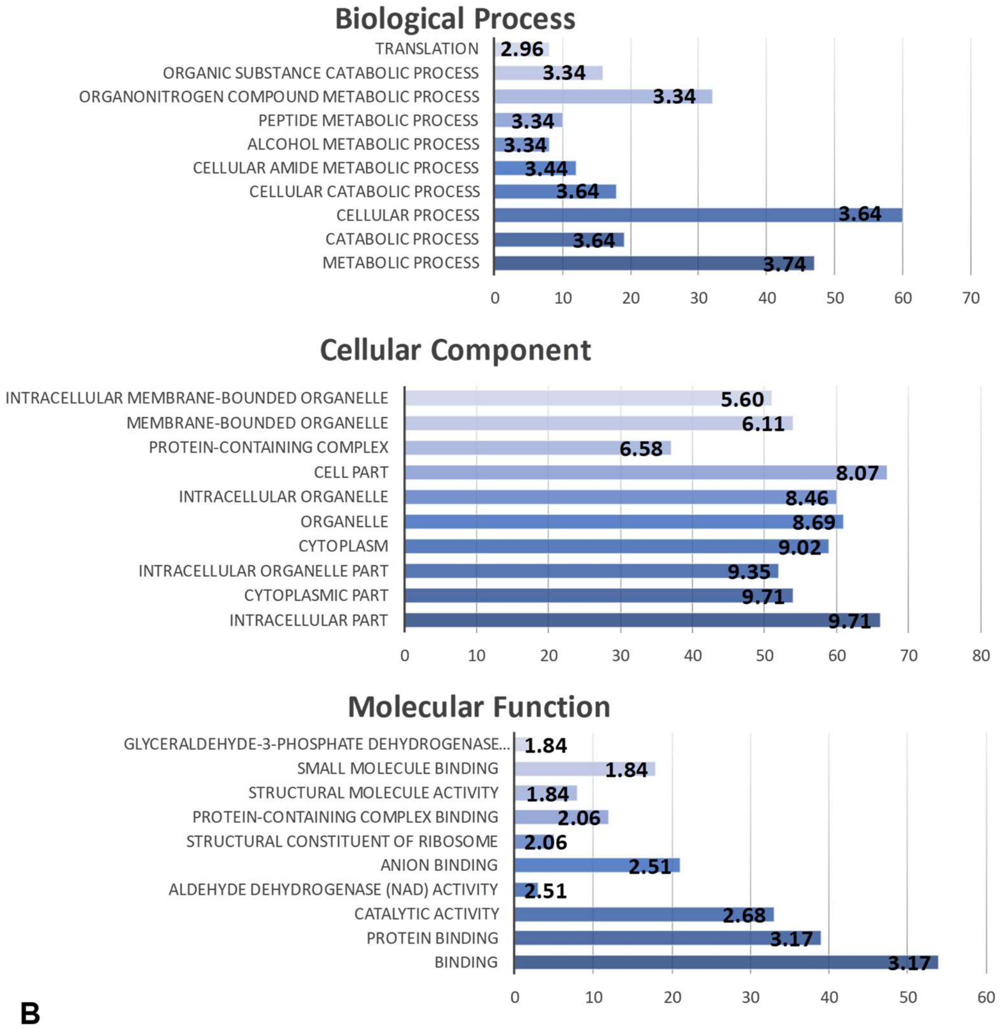

B

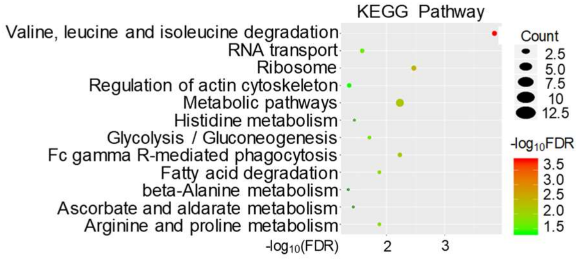

Figure S5. Soyasaponin II altered proteins function in macrophages after LPS/GalN. (A) Gene ontology (GO) enrichment analyses of discrepant protein between DMSO and SSII group. The number showed on each column represents $-\mathrm{Log}_{10}$ (FDR), associated with p-value indicating differential degree of proteins between DMSO and SSII groups, and the x-axis represents observed gene counts. (B) Top 12 KEGG pathways of discrepant proteins from the two groups.

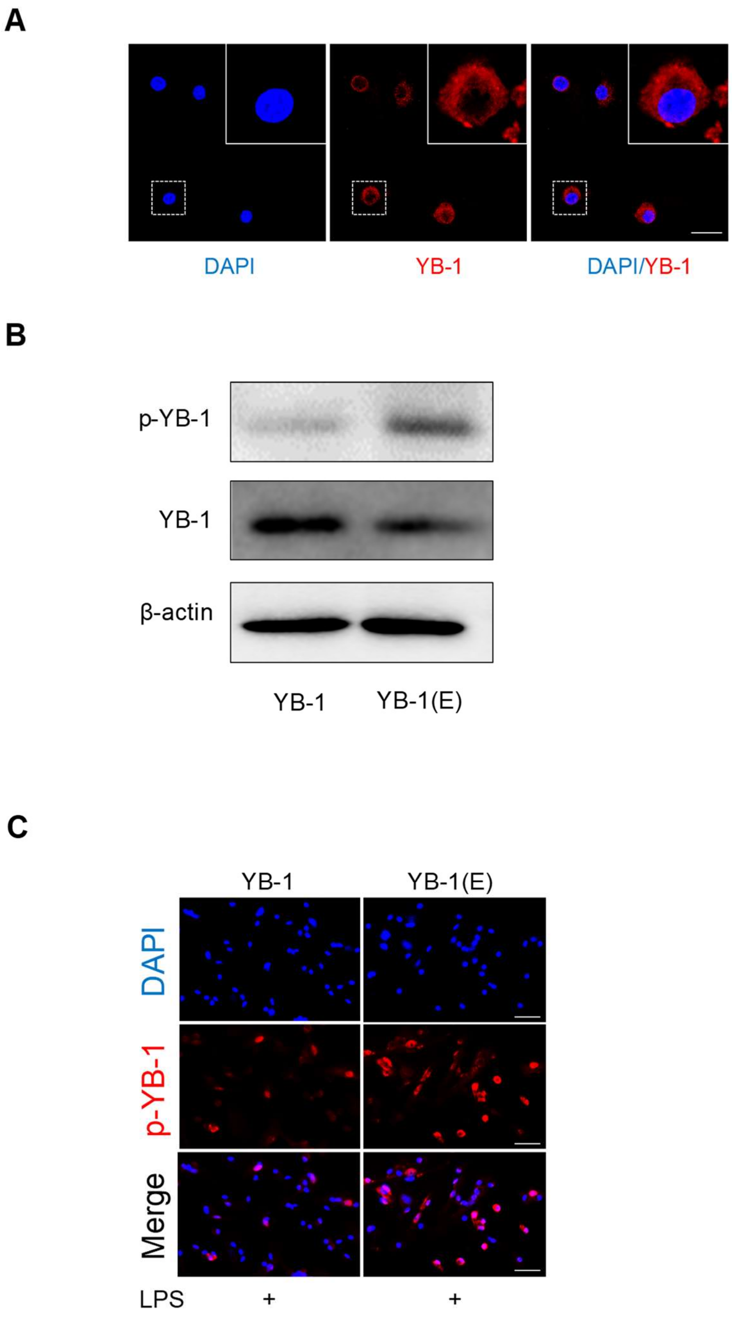

Figure S6. p-YB-1 overexpression enhanced its nuclear translocation. (A) Immunofluorescence of total YB-1 in BMDMs under normal status. (B) BMDMs were transfected with pcDNA-YB-1 mutant (Ser102 to Glu102, p-YB-1 overexpression) plasmid or pcDNA-YB-1 (control) plasmid, followed by LPS treatment for 2 h. Western blot of p-YB-1 levels in BMDMs. (C) Representative image of p-YB-1 immunofluorescence staining in BMDMs. Scale bars: 20 μm.

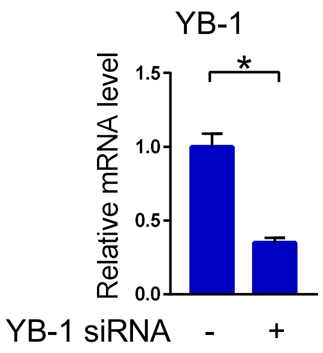

Figure S7. YB-1 siRNA sigmificantly decreased YB-1 mRNA level. BMDMs were transfected with YB-1 siRNA plasmid or negative control (NC) plasmid for 36 h. Relative mRNA level of YB-1 (n=12). *$p<0.05$.

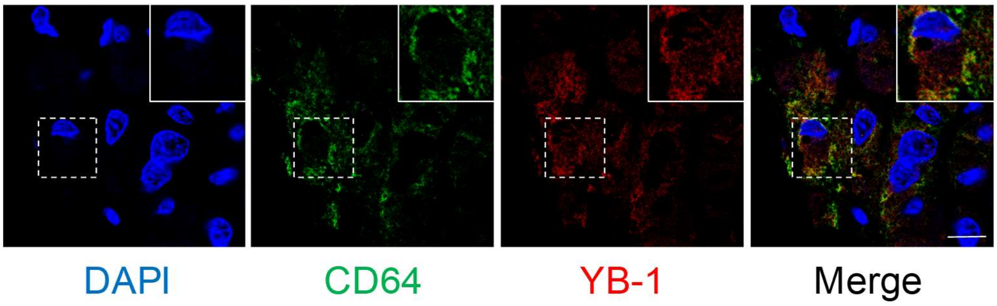

Figure S8. Immunofluorescence for CD64/total YB-1 in human (control, not ALF patient) liver section. Scale bars: 10 μm.

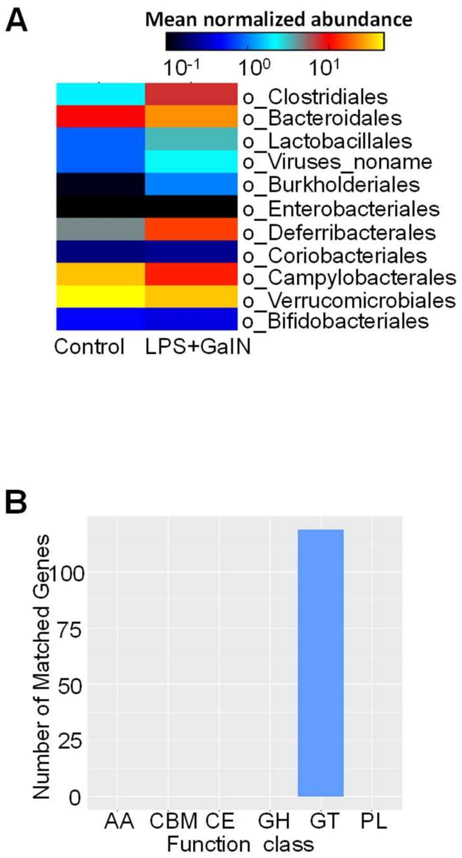

Figure S9. LPS/GalN administration altered gut microbial composition and function. C57BL/6J mice were intraperitoneally administrated with D-galactosamine (700 mg/kg) in combination with lipopolysaccharide (10 μg/kg) for 6 h. (A) Heat-Map for relative abundance of gut microbiota at order level. (B) The CAZy (Carbohydrate-Active enZYmes Database) analysis of differential gene based on metagenomics between control and LPS/GalN group. The Y-axis represents number of annotated unigenes. AA, Auxiliary Activities; CBM, Carbohydrate-Binding Modules; CE, Carbohydrate Esterases; GH, Glycoside Hydrolases; GT, Glycosyl Transferases; PL, Polysaccharide Lyases.
